# 2-Methyl-1,2,4-triazolo[4,3-*a*]pyridin-2-ium tetra­fluoroborate

**DOI:** 10.1107/S1600536813015535

**Published:** 2013-06-08

**Authors:** Siping Wei, Li Wang, Qin Wang

**Affiliations:** a Department of Medicinal Chemistry, Luzhou Medical College, Luzhou, Sichuan, 646000, People’s Republic of China

## Abstract

In the title salt, C_7_H_8_N_3_^+^·BF_4_^−^, the 1,2,4-triazolo[4,3-*a*]pyridinium cation is planar [maximum deviation of 0.016 (2) Å for all non-H atoms]. The cation and anion constitute a tight ionic pair with an F⋯N [2.911 (4) Å] inter­molecular attractive inter­action. The ionic pairs form dimers *via* stacking inter­actions between inversion-related cations, the normal distance between the cation planes being 3.376 (5) Å. The dimers are packed in stacks along the *a* axis and linked *via* C—H⋯F hydrogen bond, forming a three-dimensional network.

## Related literature

For catalytic applications of triazoliums, see: Fisher *et al.* (2006 ▶); Enders *et al.* (2006 ▶); Wurz *et al.* (2012 ▶). For the synthesis of a related compound and for related structures, see: Ma *et al.* (2008 ▶); Wei *et al.* (2009 ▶).
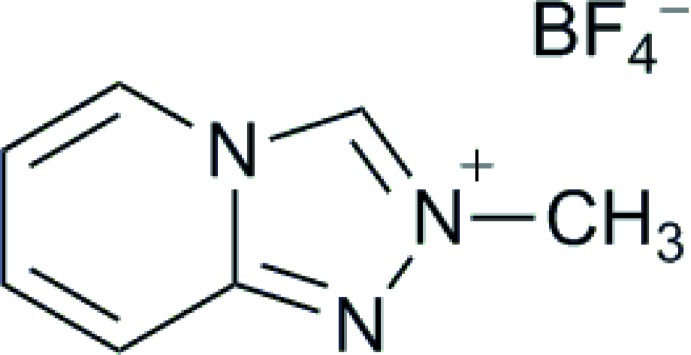


## Experimental

### 

#### Crystal data

C_7_H_8_N_3_^+^·BF_4_^−^
*M* *_r_* = 220.97
Orthorhombic, 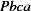

*a* = 7.1508 (10) Å
*b* = 12.3070 (18) Å
*c* = 21.431 (3) Å
*V* = 1886.0 (5) Å^3^
*Z* = 8
Mo *K*α radiation
μ = 0.15 mm^−1^
*T* = 296 K
0.30 × 0.20 × 0.20 mm

#### Data collection

Oxford Diffraction Xcalibur Eos diffractometer
Absorption correction: multi-scan (*CrysAlis PRO*; Oxford Diffraction, 2010 ▶) *T*_min_ = 0.956, *T*_max_ = 0.970
14891 measured reflections
1903 independent reflections
1616 reflections with *I* > 2σ(*I*)
*R*_int_ = 0.027

#### Refinement

*R*[*F* ^2^ > 2σ(*F* ^2^)] = 0.077
*wR*(*F* ^2^) = 0.245
*S* = 1.05
1903 reflections
137 parameters
H-atom parameters constrained
Δρ_max_ = 0.74 e Å^−3^
Δρ_min_ = −0.50 e Å^−3^

### 

Data collection: *CrysAlis PRO* (Oxford Diffraction, 2010 ▶); cell refinement: *CrysAlis PRO*; data reduction: *CrysAlis PRO*; program(s) used to solve structure: *SHELXS97* (Sheldrick, 2008 ▶); program(s) used to refine structure: *SHELXL97* (Sheldrick, 2008 ▶); molecular graphics: *OLEX2* (Dolomanov *et al.*, 2009 ▶); software used to prepare material for publication: *OLEX2*.

## Supplementary Material

Crystal structure: contains datablock(s) luo7, I. DOI: 10.1107/S1600536813015535/kq2005sup1.cif

Structure factors: contains datablock(s) I. DOI: 10.1107/S1600536813015535/kq2005Isup2.hkl

Supplementary material file. DOI: 10.1107/S1600536813015535/kq2005Isup3.cml

Additional supplementary materials:  crystallographic information; 3D view; checkCIF report

## Figures and Tables

**Table 1:** Hydrogen-bond geometry (Å, °)

*D*—H⋯*A*	*D*—H	H⋯*A*	*D*⋯*A*	*D*—H⋯*A*
C1—H1⋯F4^i^	0.93	2.18	3.086 (3)	165
C5—H5⋯F2^ii^	0.93	2.38	3.169 (4)	143
C6—H6⋯F1^iii^	0.93	2.53	3.301 (5)	141

Symmetry codes: (i) 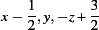; (ii) 
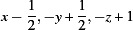
; (iii) 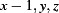.

**Fig. 1.**
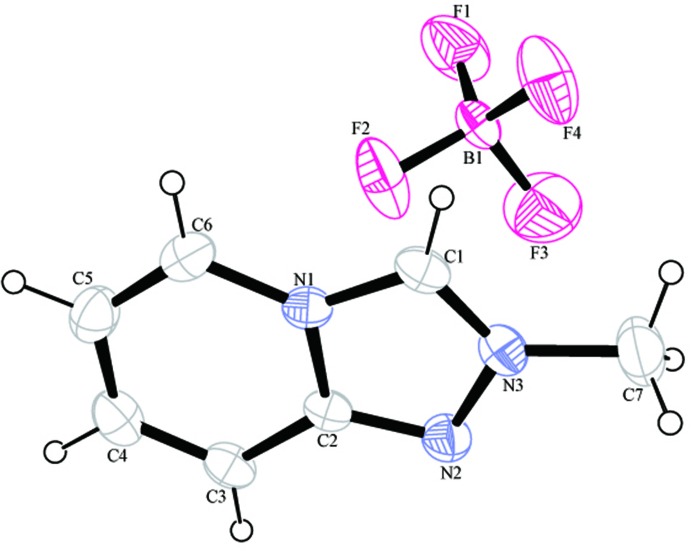
The molecular structure of (I), showing 50% probability displacement ellipsoids and the atomic numbering.

## supplementary crystallographic information

### Comment

Recently, triazolium salts which can used as carbene precursors are widely using in organic catalysis for the formation of C—C bond reactions, such as benzoin reactions, Stetter reactions and Diels-Alder reactions (Fisher *et al.* 2006; Enders *et al.* 2006; Wurz *et al.* 2012), because of their good stability and excellent catalytic performance. Most research show that bicyclic 1,2,4-triazole carbene have excellent catalytic activity because they have weaker nucleophility than that of thiazole and imidazole carbene.

The crystal structure of the title compound shows that this salt containing the 1,2,4-triazolo[4,3-*a*]pyridinium cation and tetrafluoroborate anion. The cation adopts the planar structure (r.m.s. deviation is 0.009 Å). The cation and anion constitute a tight ionic pair by the N···F (2.911 (4) Å) intermolecular attractive interaction. The ionic pairs form centrosymmetrical dimers *via* the intermolecular stacking interactions between cations (the distance between the cation planes within the dimer is 3.376 (5) Å). The dimers are packed in stacks along the *a* axis and linked into three-dimensional framework by the C—H···F hydrogen bonds.

### Experimental

The title compound was prepared according to the method of Ma *et al.* (Ma *et al.*, 2008) and Wei *et al.* (Wei *et al.*, 2009). A flame-dried round-bottomed flask equipped with a reflux condenser was charged with trimethyloxonium tetrafluoroborate (0.88 g, 6 mmol), 1-(pyridin-2-yl)hydrazine (0.55 g, 5 mmol), and chlorobenzene (20 ml). The mixture was then stirred for 30 min, followed by addition of trimethyl orthoformate (1.65 ml, 15 mmol). After being heated at 110 °C for 10 h, the reaction mixture was concentrated *in vacuo*. The resulting residue was recrystallized from acetone to give 2-Methylpyrido[1,2-*a*][1,2,4]-triazol-2-ium tetrafluoroborate as colorless crystal in 88% yield. Mp 171 - 173 °C. ^1^H NMR (400 MHz, CD_3_OD) δ 4.40 (s, 3 H), 7.44 (t, *J* = 7.2 Hz, 1 H), 7.85 - 7.88 (m, 1 H), 7.96 (d, *J* = 9.6 Hz, 1 H), 8.75 (d, *J* = 7.8 Hz, 1 H). ^13^C NMR (100 MHz, CD_3_OD) δ 40.7, 116.4, 119.8, 127.0, 134.9, 149.5. MS (ESI^+^) *m/z* 133 [*M* - BF_4_ - H]^+^. Anal. Calcd for C_7_H_8_BF_4_N_3_: C, 38.05; H, 3.65; N, 19.02. Found: C, 37.96; H, 3.50; N, 19.25. Colourless crystals suitable for X-ray structural determination were grown by slow evaporation of a solution of the title compound in a petroleum ether/actone mixture (1:1, *v*/*v*) at room temperature.

### Refinement

All H atoms were positioned geometrically and refined in the riding model approximation with C—H = 0.93 (CH_3_) or 0.96 (CH) Å.

BF_4_^-^ is a tetrahedral anion, the central boron atom is surrounded by four neighboring fluorine atoms. Consequently, the thermal movement of boron atom is limited, but not for the ending fluorine atoms. Therefore, the *U*_eq_ for the boron atom is low as compared to the neighboring fluorine atoms.

There are two relatively high positive peaks of 0.74 and 0.53 e/Å^3^ near the fluorine atoms of the BF_4_^-^ anion that indicate a slight disorder of the anion. However, due to the low contribution of the second component it was neglected.

### Crystal data

**Table:**

C_7_H_8_N_3_^+^·BF_4_^−^	*F*(000) = 896
*M**_r_* = 220.97	*D*_x_ = 1.556 Mg m^−^^3^
Orthorhombic, *P**b**c**a*	Mo *K*α radiation, λ = 0.71073 Å
*a* = 7.1508 (10) Å	µ = 0.15 mm^−^^1^
*b* = 12.3070 (18) Å	*T* = 296 K
*c* = 21.431 (3) Å	Block, colourless
*V* = 1886.0 (5) Å^3^	0.30 × 0.20 × 0.20 mm
*Z* = 8	

### Data collection

**Table:**

Oxford Diffraction Xcalibur Eos diffractometer	1903 independent reflections
Radiation source: fine-focus sealed tube	1616 reflections with *I* > 2σ(*I*)
Graphite monochromator	*R*_int_ = 0.027
Detector resolution: 16.0874 pixels mm^-1^	θ_max_ = 26.4°, θ_min_ = 1.9°
ω scans	*h* = −8→8
Absorption correction: multi-scan (*CrysAlis PRO*; Oxford Diffraction, 2010)	*k* = −15→15
*T*_min_ = 0.956, *T*_max_ = 0.970	*l* = −26→26
14891 measured reflections	

### Refinement

**Table:**

Refinement on *F*^2^	Primary atom site location: structure-invariant direct methods
Least-squares matrix: full	Secondary atom site location: difference Fourier map
*R*[*F*^2^ > 2σ(*F*^2^)] = 0.077	Hydrogen site location: inferred from neighbouring sites
*wR*(*F*^2^) = 0.245	H-atom parameters constrained
*S* = 1.05	*w* = 1/[σ^2^(*F*_o_^2^) + (0.1597*P*)^2^ + 1.099*P*] where *P* = (*F*_o_^2^ + 2*F*_c_^2^)/3
1903 reflections	(Δ/σ)_max_ < 0.001
137 parameters	Δρ_max_ = 0.74 e Å^−^^3^
0 restraints	Δρ_min_ = −0.50 e Å^−^^3^

### Special details

**Table:**

Experimental. Absorption correction: *CrysAlis PRO*, Agilent Technologies, Version 1.171.35.19, empirical absorption correction using spherical harmonics, implemented in SCALE3 ABSPACK scaling algorithm.
Geometry. All s.u.'s (except the s.u. in the dihedral angle between two l.s. planes) are estimated using the full covariance matrix. The cell s.u.'s are taken into account individually in the estimation of s.u.'s in distances, angles and torsion angles; correlations between s.u.'s in cell parameters are only used when they are defined by crystal symmetry. An approximate (isotropic) treatment of cell s.u.'s is used for estimating s.u.'s involving l.s. planes.
Refinement. Refinement of *F*^2^ against ALL reflections. The weighted *R*-factor *wR* and goodness of fit *S* are based on *F*^2^, conventional *R*-factors *R* are based on *F*, with *F* set to zero for negative *F*^2^. The threshold expression of *F*^2^ > σ(*F*^2^) is used only for calculating *R*-factors(gt) *etc*. and is not relevant to the choice of reflections for refinement. *R*-factors based on *F*^2^ are statistically about twice as large as those based on *F*, and *R*- factors based on ALL data will be even larger.

### Fractional atomic coordinates and isotropic or equivalent isotropic displacement parameters (Å^2^)

**Table:**

	*x*	*y*	*z*	*U*_iso_*/*U*_eq_	
F1	0.7647 (5)	0.2546 (3)	0.66913 (12)	0.1094 (10)	
F2	0.5247 (4)	0.3344 (2)	0.61787 (11)	0.1026 (10)	
F3	0.7464 (5)	0.4320 (3)	0.66199 (16)	0.1321 (13)	
F4	0.5444 (4)	0.3483 (3)	0.72252 (10)	0.1119 (10)	
N1	0.1517 (3)	0.41555 (15)	0.59033 (9)	0.0398 (5)	
N2	0.3053 (3)	0.57217 (18)	0.59455 (10)	0.0500 (6)	
N3	0.2503 (3)	0.53825 (19)	0.65195 (10)	0.0500 (6)	
C1	0.1599 (4)	0.4458 (2)	0.65054 (11)	0.0476 (6)	
H1	0.1110	0.4084	0.6845	0.057*	
C2	0.2444 (3)	0.49487 (18)	0.55689 (11)	0.0395 (6)	
C3	0.2603 (3)	0.4838 (2)	0.49127 (12)	0.0486 (7)	
H3	0.3203	0.5364	0.4674	0.058*	
C4	0.1859 (4)	0.3948 (2)	0.46456 (13)	0.0564 (8)	
H4	0.1952	0.3859	0.4216	0.068*	
C5	0.0940 (5)	0.3147 (2)	0.50043 (15)	0.0599 (8)	
H5	0.0454	0.2538	0.4805	0.072*	
C6	0.0751 (4)	0.3243 (2)	0.56234 (15)	0.0544 (7)	
H6	0.0130	0.2718	0.5856	0.065*	
C7	0.2944 (6)	0.6039 (3)	0.70666 (15)	0.0775 (10)	
H7A	0.2205	0.6690	0.7061	0.116*	
H7B	0.4247	0.6227	0.7061	0.116*	
H7C	0.2671	0.5632	0.7438	0.116*	
B1	0.6389 (4)	0.3369 (3)	0.66829 (12)	0.0498 (7)	

### Atomic displacement parameters (Å^2^)

**Table:**

	*U*^11^	*U*^22^	*U*^33^	*U*^12^	*U*^13^	*U*^23^
F1	0.126 (2)	0.124 (2)	0.0777 (16)	0.0613 (17)	−0.0112 (13)	0.0018 (12)
F2	0.0934 (17)	0.150 (2)	0.0640 (13)	0.0390 (15)	−0.0291 (11)	−0.0343 (13)
F3	0.121 (3)	0.133 (2)	0.142 (3)	−0.044 (2)	−0.0029 (18)	0.0240 (19)
F4	0.0957 (18)	0.191 (3)	0.0492 (12)	−0.0054 (18)	0.0215 (11)	−0.0277 (13)
N1	0.0366 (10)	0.0397 (10)	0.0431 (10)	0.0044 (8)	0.0029 (7)	0.0056 (7)
N2	0.0533 (12)	0.0509 (12)	0.0456 (12)	−0.0067 (9)	0.0023 (9)	0.0053 (9)
N3	0.0536 (13)	0.0574 (13)	0.0389 (11)	0.0006 (10)	0.0010 (8)	0.0016 (9)
C1	0.0477 (14)	0.0564 (14)	0.0386 (12)	0.0048 (11)	0.0060 (9)	0.0102 (10)
C2	0.0329 (10)	0.0455 (12)	0.0401 (13)	0.0038 (8)	0.0022 (8)	0.0082 (9)
C3	0.0420 (13)	0.0621 (15)	0.0416 (13)	0.0070 (11)	0.0045 (10)	0.0087 (11)
C4	0.0525 (15)	0.0716 (18)	0.0452 (14)	0.0193 (13)	0.0003 (11)	−0.0086 (12)
C5	0.0613 (17)	0.0502 (15)	0.0682 (18)	0.0065 (12)	−0.0054 (14)	−0.0129 (12)
C6	0.0544 (15)	0.0413 (12)	0.0674 (17)	−0.0007 (11)	0.0006 (13)	0.0023 (11)
C7	0.096 (3)	0.088 (2)	0.0488 (16)	−0.0100 (19)	−0.0080 (17)	−0.0151 (15)
B1	0.0478 (16)	0.0710 (19)	0.0305 (12)	0.0042 (13)	0.0016 (10)	0.0002 (11)

### Geometric parameters (Å, º)

**Table:**

F1—B1	1.356 (4)	C2—C3	1.418 (4)
F2—B1	1.355 (3)	C3—C4	1.345 (4)
F3—B1	1.407 (5)	C3—H3	0.9300
F4—B1	1.352 (3)	C4—C5	1.412 (5)
N1—C1	1.344 (3)	C4—H4	0.9300
N1—C2	1.380 (3)	C5—C6	1.339 (4)
N1—C6	1.386 (3)	C5—H5	0.9300
N2—C2	1.322 (3)	C6—H6	0.9300
N2—N3	1.357 (3)	C7—H7A	0.9600
N3—C1	1.309 (4)	C7—H7B	0.9600
N3—C7	1.459 (4)	C7—H7C	0.9600
C1—H1	0.9300		
			
C1—N1—C2	106.3 (2)	C6—C5—C4	121.7 (3)
C1—N1—C6	131.1 (2)	C6—C5—H5	119.2
C2—N1—C6	122.5 (2)	C4—C5—H5	119.2
C2—N2—N3	103.7 (2)	C5—C6—N1	117.4 (2)
C1—N3—N2	112.9 (2)	C5—C6—H6	121.3
C1—N3—C7	127.4 (2)	N1—C6—H6	121.3
N2—N3—C7	119.7 (3)	N3—C7—H7A	109.5
N3—C1—N1	106.5 (2)	N3—C7—H7B	109.5
N3—C1—H1	126.7	H7A—C7—H7B	109.5
N1—C1—H1	126.7	N3—C7—H7C	109.5
N2—C2—N1	110.5 (2)	H7A—C7—H7C	109.5
N2—C2—C3	130.4 (2)	H7B—C7—H7C	109.5
N1—C2—C3	119.1 (2)	F4—B1—F2	112.8 (3)
C4—C3—C2	118.0 (2)	F4—B1—F1	113.4 (3)
C4—C3—H3	121.0	F2—B1—F1	113.2 (3)
C2—C3—H3	121.0	F4—B1—F3	105.6 (3)
C3—C4—C5	121.4 (3)	F2—B1—F3	105.8 (3)
C3—C4—H4	119.3	F1—B1—F3	105.1 (3)
C5—C4—H4	119.3		
			
C2—N2—N3—C1	−0.2 (3)	C1—N1—C2—C3	179.2 (2)
C2—N2—N3—C7	179.7 (3)	C6—N1—C2—C3	0.8 (3)
N2—N3—C1—N1	−0.3 (3)	N2—C2—C3—C4	179.2 (3)
C7—N3—C1—N1	179.8 (3)	N1—C2—C3—C4	−0.9 (3)
C2—N1—C1—N3	0.7 (3)	C2—C3—C4—C5	0.2 (4)
C6—N1—C1—N3	179.0 (2)	C3—C4—C5—C6	0.7 (4)
N3—N2—C2—N1	0.7 (3)	C4—C5—C6—N1	−0.9 (4)
N3—N2—C2—C3	−179.5 (2)	C1—N1—C6—C5	−177.9 (3)
C1—N1—C2—N2	−0.9 (2)	C2—N1—C6—C5	0.1 (4)
C6—N1—C2—N2	−179.3 (2)		

### Hydrogen-bond geometry (Å, º)

**Table:**

*D*—H···*A*	*D*—H	H···*A*	*D*···*A*	*D*—H···*A*
C1—H1···F4^i^	0.93	2.18	3.086 (3)	165
C5—H5···F2^ii^	0.93	2.38	3.169 (4)	143
C6—H6···F1^iii^	0.93	2.53	3.301 (5)	141

Symmetry codes: (i) *x*−1/2, *y*, −*z*+3/2; (ii) *x*−1/2, −*y*+1/2, −*z*+1; (iii) *x*−1, *y*, *z*.
